# Linguistic validation, validity and reliability of the British English versions of the Disabilities of the Arm, Shoulder and Hand (DASH) questionnaire and QuickDASH in people with rheumatoid arthritis

**DOI:** 10.1186/s12891-018-2032-8

**Published:** 2018-04-16

**Authors:** Alison Hammond, Yeliz Prior, Sarah Tyson

**Affiliations:** 1 Centre for Health Sciences Research (OT), L701 Allerton, University of Salford, Frederick Road, Salford, M6 6PU UK; 2 Division of Nursing, Midwifery & Social Work, University of Manchester, Manchester, UK

**Keywords:** Patient reported outcomes, Upper limb assessment, Rehabilitation, Rheumatoid arthritis

## Abstract

**Background:**

Although the Disabilities of the Arm, Shoulder and Hand (DASH) questionnaire is widely used in the UK, no British English version is available. The aim of this study was to linguistically validate the DASH into British English and then test the reliability and validity of the British English DASH, (including the Work and Sport/Music DASH) and QuickDASH, in people with rheumatoid arthritis (RA).

**Methods:**

The DASH was forward translated, reviewed by an expert panel and cognitive debriefing interviews undertaken with 31 people with RA. Content validity was evaluated using the ICF Core Set for RA. Participants with RA (*n* = 340) then completed the DASH, Health Assessment Questionnaire (HAQ), Short Form Health Survey v2 (SF36v2) and Measure of Activity Performance of the Hand (MAPHAND). We examined internal consistency and concurrent validity for the DASH, Work and Sport/Music DASH modules and QuickDASH. Participants repeated the DASH to assess test-retest reliability.

**Results:**

Minor wording changes were made as required. The DASH addresses a quarter of Body Function and half of Activities and Participation codes in the ICF RA Core Set. Internal consistency for DASH scales were consistent with individual use (Cronbach’s alpha = 0.94–0.98). Concurrent validity was strong with the HAQ (r_s_ = 0.69–0.91), SF36v2 Physical Function (r_s_ = − 0.71 - − 0.85), Bodily Pain (r_s_ = − 0.71 - − 0.74) scales and MAPHAND (r_s_ = 0.71–0.93). Test-retest reliability was good (r_s_ = 0.74–0.95).

**Conclusions:**

British English versions of the DASH, QuickDASH and Work and Sport/Music modules are now available to evaluate upper limb disabilities in the UK. The DASH, QuickDASH, Work and Sport/Music modules are reliable and valid to use in clinical practice and research with British people with RA.

**Electronic supplementary material:**

The online version of this article (10.1186/s12891-018-2032-8) contains supplementary material, which is available to authorized users.

## Background

Rheumatoid arthritis (RA) impacts on hand and upper limb function. Within two years of diagnosis, 93% of people with RA report hand pain, 82% hand stiffness, 73% hand muscle weakness, 70% have at least one hand impairment and 50% experience shoulder joint tenderness and have reduced shoulder function [1–3]. Rehabilitation therefore includes maintaining and improving hand and upper limb function [4]. Using reliable, valid outcome measures is important to ensure problems are accurately identified and treatment benefits demonstrated.

The Disabilities of the Arm, Shoulder and Hand (DASH) questionnaire is a widely used patient reported outcome measure (PROM) of upper limb function used in musculoskeletal conditions [5]. Its purpose is to detect upper limb disorders of differing severity, assess changes over time and evaluate outcomes of interventions [6]. It is one of the best upper limb measures clinimetrically [7, 8]. The QUICKDASH, a shorter, more quickly administered version derived from the DASH, was developed using Rasch analysis [9–11]. Both also include optional modules for those whose jobs require a lot of upper limb performance (WORKDASH) and for sports people and musicians (sports and music: SPAMDASH).

The DASH was originally published in Canadian/North American English. Outcome measures should be linguistically validated (i.e. translated and culturally adapted) into the language of the target country and psychometrically tested with target population(s) before being used in that country [12, 13]. There are English versions of the DASH for Australia, Hong Kong and South Africa [14] but a British English version has not yet been linguistically validated and psychometrically tested in the United Kingdom (UK). Currently, the Canadian/North American English version is being used in rheumatology clinical practice and research. Whilst much of the North American English DASH is understandable to British English speakers, clinicians and patients regularly comment that some activities included are: unclear, e.g. “yard work”; not in common usage e.g. “transportation”; infrequently performed in the UK, e.g. “wash walls.” Additionally, some phrases and sentences could be shortened to reflect Plain English usage. Consequently, a British English version is required that is then psychometrically tested in populations it is commonly used with.

The DASH consists of 30 items evaluating upper limb-related activities, participation and symptoms [11]. There has been some debate as to whether the DASH is unidimensional. Factor analysis of the original Canadian/North American [11] and also Dutch [15], Japanese [16] and Chinese [17] versions of the DASH identified a single factor and thus all items can be summed to form a total score. However, studies using factor and /or Rasch analysis with the Canadian/North American DASH in the UK identified two factors [18] while the French [19], Italian [20] and Canadian /North American [21] versions revealed three factors. Psychometric testing of measures should include a combination of classical testing and item response theory (e.g. Rasch analysis) to establish psychometric properties, including unidimensionality [22].

The overall aims of this study were to: linguistically validate the DASH into British English; investigate content validity of the DASH in RA; and evaluate the psychometrics of the British English DASH and QuickDASH amongst people with RA in the UK. The psychometrics assessed were: concurrent and discriminant validity, internal consistency, test retest reliability, sensitivity to change, compliance (amount of missing data) and floor and ceiling effects of the British English DASH and QuickDASH amongst British people with RA.

Alongside this, we also investigated construct validity of the British English DASH and QuickDASH using Rasch analysis. This is reported separately [Prodinger B, Hammond A, Tennant A, Prior Y, Tyson S. Deconstructing the Disabilities of the Arm, Shoulder and Hand (DASH) and QuickDASH in Rheumatoid Arthritis, submitted].

## Methods

Ethical approval was obtained from the National Research Ethics Service Committee North West - Greater Manchester North (12/NW/0841) and the University of Salford’s School of Health Sciences Ethics Panel. All participants provided written, informed consent.

### Participants

Participants were recruited: by research nurses screening for eligibility in 17 Rheumatology out-patient clinics (either in clinic or identified from departmental databases); and from amongst participants in a previous outcome measure study we conducted, who had consented to be contacted for future studies. All were recruited from the same Rheumatology out-patient clinics originally and with whom eligibility was re-checked prior to consent. Participants were eligible if they: had a confirmed diagnosis of RA; were able to read, write and understand English; and had not (or were not about to) altered their disease-modifying medication regimen in the last three months (which could affect test-retest reliability).

### Linguistic and cross-cultural validation

The adaptation procedures devised by the Institute of Work and Health for DASH translation were followed [23]. This consists of six steps:

1. *forward translation*: two translators (AH: a rheumatology rehabilitation researcher familiar with the DASH) and a non-health professional unfamiliar with the DASH (JG: an experienced teacher) independently reviewed the DASH to identify any words that needed to be changed into British English (e.g. transportation is termed transport) and use of Plain English (i.e. simplifying words and phrases).
2. *translation synthesis*: an independent recorder assisted the two translators agreeing any recommended changes
3. *backward translation*: was not required as the translation was into another form of English.
4. *expert committee review*: The committee included: the two translators (AH, JG); synthesis recorder (YP); an experienced Rheumatology occupational therapist familiar with using the DASH (AJ); an English language expert (GMcL); a Canadian English-speaking researcher (KH); and an experienced outcome measures researcher (ST). The committee discussed the synthesised translation, made additional recommendations and agreed and approved the wording of the draft British English DASH. This process ensures semantic, idiomatic, experiential and conceptual equivalence.
5. *field testing of the adapted DASH with people with RA*: Cognitive debriefing interviews are commonly used during PROM development to investigate the appropriateness of items and to gain insight into participants’ understanding of the content of measures [12, 24]. Participants with RA were recruited from four Rheumatology out-patient clinics. They completed the draft British English DASH (including the two optional modules if applicable) in their own time and were interviewed within two weeks about the relevance and comprehensibility of items. The results were discussed with the expert committee and, if necessary, further changes in wording made and the final British English DASH agreed. Finally, the Flesch Reading Ease score was calculated using Microsoft Word to check its readability is similar to the original DASH.
  Content validity: we systematically linked the DASH items (and sub-items, where applicable) to the International Classification of Functioning, Disability and Health (ICF) Core Set for RA [25, 26]. DASH items have previously been linked to the ICF [27].
6. *psychometric testing of the British English DASH with people with RA in the UK.*
  After each of steps 4, 5 and 6 reports were sent to the Institute of Work and Health for translation approval before proceeding to the next step [23].

### Psychometric testing procedures

Participants were mailed a questionnaire booklet which collected data to describe the recruited population: demographic and disease data: age, gender, marital, educational and employment status, disease duration and RA disease-modifying medication as well as the measures described below. Two to three weeks later, participants were mailed the British English DASH to complete at home a second time (to evaluate test-retest reliability). Two reminders were sent for each mailing, as necessary.

### Measurement instruments

#### The British English DASH

The DASH consists of 30 items, measured using five-point Likert scales (1–5): 21 regarding daily activity; five regarding symptoms; three about participation (the impact of the condition on daily life); and one about confidence in abilities [28]. The QUICKDASH was derived from the DASH and consists of 11 items (six of daily activity ability; two about symptoms (pain and tingling); and three about participation) [11]. The two optional modules (SPAM- and WORK-DASH) were also included.

#### The medical outcomes survey 36 item short-from health survey version 2 (SF36v2)

From which sub-scales of Physical Function, Bodily Pain and Vitality (fatigue) scales were selected [29, 30]. QualityMetric Health Outcomes™ Scoring Software 4.5 was used to manage missing SF36v2 data and calculate norm-based scores converted to 0–100 scale for each sub-scale [31]. Lower scores denote worse health states.

#### The health assessment questionnaire (HAQ)

Indicates ability to perform 20 daily activities rated on a 0–3 scale (0 = not at all difficult; 3 = unable to do) [32], scored using the HAQ20 method, in which the total score is obtained by summing all 20 items (0–20 = mild; 21–40 = moderate; 41–60 = severe disability) [33, 34]. This method was used as the HAQ20 does not weight items worse if an assistive device is used, as occurs when normally scoring the HAQ. Higher scores denote greater activity limitations.

#### The hand HAQ

Seven items of upper limb function derived from the HAQ (i.e. Dressing; Cutting meat/food; Lifting a full cup or glass; Opening a new milk carton; Opening car doors; Opening jars which have been previously opened; Turning taps on and off [35]. The score is the sum of the seven items, with higher scores denoting greater activity limitations.

#### The British English measure of activity performance of the hand (MAP-HAND)

Eighteen items of activity ability requiring hand use, each measured on a 0–3 scale (0 = not at all difficult; 3 = unable to do) [36, 37]. The total score is obtained by summing the 18 items, with higher scores denoting greater activity limitations.

#### Symptom 10-point numeric rating scales (NRS)

Evaluating: hand pain on activity; and self-reported disease activity level, general pain at rest, general pain on movement, stiffness, movement limitations, from the Evaluation of Daily Activity Questionnaire [38].

#### RA quality of life scale (RAQOL)

Thirty items about quality of life (QoL) answered yes (=1) or no (=0), with yes items summed to give a total score. Higher scores indicate worse QoL [39].

#### Perceived change in health status

At Test 2 only, this was measured using a 5-point NRS by asking “*Overall, how much is your arthritis troubling you now compared to when you last completed this questionnaire?”* (1 = much less; 2 = somewhat less; 3 = about the same; 4 = somewhat more; 5 = much more).

We hypothesised that there would be strong correlations between the four DASH scales and these measures.

### Sample size

As Rasch analysis was also being used to assess construct validity of the British English DASH, a sample size of at least 250 was recruited [Prodinger B, Hammond A, Tennant A, Prior Y, Tyson S. Deconstructing the Disabilities of the Arm, Shoulder and Hand (DASH) and QuickDASH in Rheumatoid Arthritis, submitted]. This number was determined from the need to ensure a uniform distribution of patients across the construct of upper limb function, so that the precision of the estimate of both persons and items, across the construct, remains similar [40]. At least 79 sets of repeated responses were required to demonstrate that a test-retest correlation of 0.7 differs from a background correlation (constant) of 0.45, with 90% power at the 1% significance level. A test-retest correlation of 0.7 is deemed a minimum acceptable level [41].

### Statistical analyses

Rasch analyses of both the DASH and QUICKDASH indicated that, using a testlet approach taking account of local dependency, both can be considered as unidimensional and total raw scores, standardised to 0–100, can therefore be used [Prodinger B, Hammond A, Tennant A, Prior Y, Tyson S. Deconstructing the Disabilities of the Arm, Shoulder and Hand (DASH) and QuickDASH in Rheumatoid Arthritis, submitted]. DASH and QuickDASH standardised scores can be converted to a Rasch metric interval scale when required for parametric analyses [Prodinger B, Hammond A, Tennant A, Prior Y, Tyson S. Deconstructing the Disabilities of the Arm, Shoulder and Hand (DASH) and QuickDASH in Rheumatoid Arthritis, submitted].

For both the DASH and QUICKDASH, standardised (0–100) scores are calculated by:

$$ \mathrm{DASH}\ \mathrm{DISABILITY}/\mathrm{SYMPTOM}\ \mathrm{SCORE}=\frac{\left[\left(\mathrm{sum}\ \mathrm{of}\ \mathrm{n}\ \mathrm{responses}\right)\hbox{--} 1\right]}{\mathrm{n}}\times 25 $$

(where n is the number of completed responses). A higher score represents worse ability/symptoms. The DASH score cannot be calculated if there are more than three missing items, nor the QUICKDASH if more than one missing item**.**

The WORK- and SPAM-DASH were scored by: adding the assigned values for each response, dividing by 4 (number of items); subtracting 1; and multiplying by 25 to convert to a 0–100 scale. Optional module scores cannot be calculated if there are missing items.

The Statistical Package for the Social Sciences v20 was used for analyses [42], apart from linear weighted kappas, calculated using MedCalc [43]. As all measures consist of ordinal data, non-parametric statistical tests were used to assess the psychometrics.

#### Concurrent validity

Of the four DASH scores was assessed using Spearman’s correlations with measures of related constructs (i.e. SF36v2 sub-scales, HAQ20, Hand HAQ, MAP-HAND, RAQOL, and symptom NRSs). Correlations of 0.8–1.00 were deemed very strong; 0.6–0.79 strong; 0.4–0.59 moderate; 0.20–0.39 weak; and 0–0.19 are very weak [44].

#### Discriminant validity

Was assessed using Kruskal-Wallis tests to evaluate differences in scores between participants with different degrees of disease activity, using the disease activity NRS (low disease activity = 0–3; moderate = 4–6; high = 7–10).

#### Internal consistency

Was assessed using Cronbach’s alpha. Results of ≥0.8 were deemed good to excellent [44]. A value of ≥0.85 is consistent with individual use and > 0.7 with group-level use.

#### Test-retest reliability

Was assessed, in those stating their condition was “the same” at Test 2, using Spearman’s correlations and intra-class correlation coefficients (ICC (2,1): two-way random consistency, average measures model). An ICC ≥ 0.75 was considered excellent [45]. Reliability of individual DASH items was calculated using linear weighted kappa. Levels of agreement are interpreted as < 0.20 = poor; 0.21–0.40 = fair; 0.41–0.60 = moderate; 0.61–0.80 = good; 0.81–1.00 = very good [46].

#### Sensitivity to change

Was assessed by calculating Standard Error of Measurement (SEM) and the Minimal Detectable Change_95_ (MDC_95_) scores, i.e. a statistical estimate of the smallest detectable change corresponding to change in ability [47, 48].

The formulae used were: *SEM* = *s* √ (1 – *r*), where s = the mean and standard deviation (SD) of Test 1 and Test 2 (retest), r = the reliability coefficient for the test, i.e. Pearson’s correlation co-efficient between Test and Test 2 values. Thereafter the MDC_95_ was calculated using the formula: *MDC*_95_ = *SEM* ×  √ 2 × 1.96 [48].

#### Compliance (missing data)

The number of missing data items were reviewed to identify the percentage of the four DASH scales which could not be scored, and the commonest missing items.

#### Floor and ceiling effects

Were considered present if > 15% of participants achieved either the lowest or highest scores in the four DASH scales [49, 50].

## Results

### Steps 1 to 5: Linguistic validation and cross-cultural adaptation

The expert panel agreed several changes to simplify language: “perform” was changed to “do”; “estimate” to “guess”; “household chores” to “household jobs”; “wash floors” to “clean floors”; “put on a pullover sweater” to “put on a jumper”; “transportation” to “transport”; “using your usual technique for your work” to “doing your work in your usual way”; “using your usual technique for playing your instrument or sport” to “playing your instrument or sport in your usual way”; “yard work” to “outdoor property work” (as this was identified as meaning outdoor property maintenance in Canada); “wash walls” to “wash windows” (as the former is a rare activity and washing windows requires a similar action); and for “carry a heavy object (over 10lbs)” we added “or 5 kg” to provide a rough metric equivalent.

Cognitive debriefing interviews were conducted with 26 women and five men (see Table 1). Minor changes to clarify were suggested for seven items. Five participants were unsure whether the instruction “ability to do the following activities…” referred to ability with or without aids and adaptations, as they might answer differently using these. The panel agreed not to change instructions as these are consistent across all language versions of the DASH. For the activity items, only two raised interpretation concerns. Five interpreted “Make a bed” (item 9) as completely changing the bed linen. In British English, “make a bed” describes the daily tidying or straightening bedding and was interpreted as such by other participants. Discussion with Canadians indicated that this means the same in Canadian/North American English. Nine queried whether “manage transport needs” (item 20) referred specifically to driving, getting a lift or using public transport, as each required different levels of upper limb activity, or to multiple transport methods. Other participants interpreted this related to their own travel circumstances. For symptom severity, eight participants indicated it was difficult differentiating between “arm, shoulder or hand pain severity” (item 24), and pain severity “when you do any specific activity” (item 25) as their pain usually lasts some time without changing with different activities. However, the other participants could identify activities inducing/ exacerbating pain and thus rate these items separately. Five were unable to identify whether the “weakness in their arms, shoulder or hand” (item 27) was any different in the last week than usual, as their upper limb was constantly weak. Thirteen were unsure if they could solely attribute sleeping problems to arm, shoulder or hand pain (item 29) as they either had multiple painful joints or widespread pain, although they did answer the question. The panel discussed these items and decided not to make further changes. The Flesch Reading Ease score for the British English DASH was 62.8, i.e. similar to the Canadian DASH (61.5), indicating a reading age of 13 to 15-year olds is required [51].

**Table 1:** DASH study participant characteristics (*n* = 340)

Participant Characteristics	Cognitive debriefing Participants (*n* = 31)	Psychometric testing: Participants (*n* = 340)
Age:(Mean (SD)	63.42 (12.04)	61.96 (12.09)
Gender (M:F)	5:26	89:251
Condition duration (years) (Mean (SD):	15.71 (12.61)	14.44 (11.73)
Marital status: n (%)		
Married/living with partner	23 (74%)	241 (71%)
Living status: n (%)		
Family/significant other	24 (77%)	245 (72%)
Children living at home	4 (13%)	36 (11%)
Employment status		
Paid employment	3 (10%)	108 (32%)
Retired	22 (71%)	204 (60%)
Other	6 (19%)	28 (8%)
Education level (ISCED)		
Secondary education only	19 (61%)	182 (54%)
Current medication		
Not on DMARDs	2 (6%)	34 (10%)
Monotherapy	10 (32%)	91 (27%)
Combination therapy	10 (32%)	190 (56%)
Biologic drugs	9 (29%)	25 (7%)

#### Content validity

Using the Brief ICF Core Set for RA, the DASH addresses: 5/24 Body Functions codes, 0/13 Body Structures codes; 15/26 Activities and Participation codes; and 0/5 Environmental Factors codes. Eight items were linked to either fine hand use (d440) or hand and arm use (d445) and allocated to carrying, moving and handling, other (d449). Five DASH items were not linked to the Brief ICF Core Set: gardening (item 8); interference with social activities (item 22); tingling (item 26); weakness (item 27); and feeling less capable (item 30), as the Core Set does not include Personal Factors. (See Additional file 1: Table S1).

### Step 6: Psychometric testing

#### Participants

**Fig. 1:**
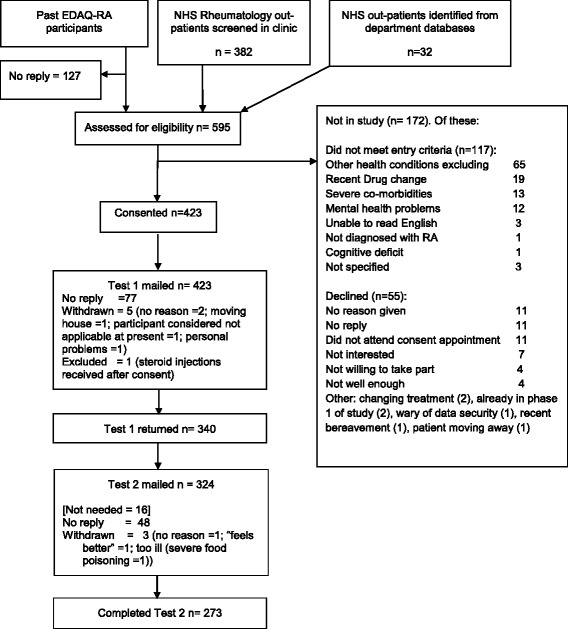
British English DASH in RA: Recruitment & Study Progress Flow Diagram. Key: DASH = Disabilities in the Arm, Shoulder and Hand questionnaire; EDAQ = Evaluation of Daily Activity Questionnaire; RA = Rheumatoid Arthritis study; NHS = National Health Service

Overall, 595 people were screened for eligibility, 423 consented and 340 returned the Test 1 questionnaire booklet and 273 the Test 2 booklet (see Fig. 1). Participant characteristics are shown in Table 1 and health status, activity limitations and quality of life measures descriptive data are shown in Table 2. The mean time between tests was 34.6 (SD 13.07) days.

**Table 2:** Descriptive data for health status measures (*n* = 340)

Health status measures (median (IQR)	Test 1 (*n* = 340)	Test 2 (*n* = 273)
DASH (range 0–100)	35.34 (18.33–56.35)	36.67 (16.95–55.00)
QuickDASH (range 0–100)	34.09 (15.91–50.0)	36.36 (18.18–56.81)
WORKDASH (0–100)	25 (6.25–43.75) (*n* = 158)	25 (0–39.06) (*n* = 118)
SPAMDASH (range 0–100)	25 (12.50–59.38) (*n* = 57)	31.25 (18.75–75.0) (*n* = 39)
Test 1 only:		
Disease activity level NRS (range 0–10)	4 (2–6)	
Pain when moving NRS (range 0–10)	5 (2–7)	
SF36v2 Bodily Pain (range 0–100)	42.24 (34.18–47.48)	
Hand pain on activity NRS (range 0–10)	4 (2–7)	
Fatigue NRS (0–10)	6 (4–8)	
SF36v2 Vitality (range 0–100)	43.69 (34.77–49.63)	
HAQ20 (0–60)	13 (4–23)	
Hand HAQ (range 0–21)	5 (1.75–10)	
MAPHAND (range 0–54)	17 (8.25–27)	
SF36v2 Physical Function (range 0–100)	36.49 (26.93–46.06)	
RAQOL (range 0–30)	10.50 (4–19)	

#### Concurrent validity

The DASH correlated strongly with all disease activity, symptom, function and quality of life measures (r_s_ = 0.61–0.99); as did the QuickDASH (r_s_ = 0.61–0.91). WORKDASH correlations were mainly strong (r_s_ = 0.53–0.80); and SPAMDASH correlations moderate to strong (r_s_ = 0.52–0.78) (see Table 3).

**Table 3:** Concurrent validity of the DASH, WORKDASH and SPAMDASH with health status, activity limitation and quality of life measures

	Disease activity NRS	Pain on movement NRS	Fatigue NRS	Hand pain on activity NRS	HAQ20	Hand HAQ	MAPHAND	RAQOL	SF36v2 Physical Function	SF36v2 Bodily Pain	SF36v2 Vitality
DASH (*n* = 340)	0.61**	0.70**	0.64**	0.75**	0.91**	0.88**	0.93**	0.80**	-0.85**	-0.74**	−0.63**
QuickDASH (*n* = 340)	0.61**	0.70**	0.65**	0.76**	0.87**	0.84**	0.91**	0.79**	−0.82**	− 0.73**	−0.62**
WORKDASH (*n* = 158)	0.54**	0.62**	0.62**	0.69**	0.80**	0.74**	0.74**	0.74**	−0.71**	−0.71**	− 0.53**
SPAMDASH (*n* = 57)	0.52**	0.55**	0.48**	0.57**	0.69**	0.60**	0.71**	0.78**	−0.74**	− 0.71**	−0.61**

*Key*: Spearman’s correlations; ** *p* < 0.001; *NRS* numeric rating scale

#### Discriminant validity

There were significant differences between the three levels of perceived disease activity for the DASH, QuickDASH, WORKDASH and SPAMDASH, with participants with higher perceived disease activity scoring worse on the DASH scales (see Table 4).

**Table 4:** Discriminant validity: DASH (*n* = 327), QuickDASH (*n* = 334), WORKDASH (*n* = 157) and SPAMDASH (*n* = 57) median (IQR) scores and differences between perceived disease activity groups

	Low disease activity (0–3)	Moderate disease activity (4–6)	High disease activity (7–10)	Chi-square	df	p
DASH	19.58 (9.58–36.32)	42.81 (27.29–58.33)	57.50 (43.33–72.50)	399.40	332	0.007
QuickDASH	15.91 (6.82–36.36)	40.91 (25.00–52.27)	56.82 (39.77–65.91)	214.00	102	0.000
WORKDASH	12.50 (0–29.69)	28.13 (18.75–50.0)	50.0 (31.25–68.75)	71.37	28	0.000
SPAMDASH	25 (0–37.5)	56.25 (31.25–87.50)	100 (75.00–100)	50.02	28	0.006

#### Internal consistency

Cronbach’s alpha values for the four DASH scales were excellent ranging from 0.94 (WORKDASH) to 0.98 (DASH) (see Table 5).

**Table 5:** Internal consistency and test-retest reliability of the DASH, QuickDASH, WORKDASH and SPAMDASH (for those reporting “the same” at Test 2)

	Cronbach’s alpha	n for test-retest	Test 1 score (median, IQR)	Test 2 score (median, IQR)	Spearman’s Correlation (r_s_)	ICC(2,1) (95% CI)
DASH	0.98	170	30.83 (15.83–55.00)	30.00 (12.50–53.33)	0.95**	0.97 (0.96,0.98)
QuickDASH	0.94	180	29.55 (13.63–47.73)	30.00 (13.63–53.41)	0.93**	0.95 (0.94,0.96)
WORKDASH	0.94	53	25.00 (6.25–37.50)	25.00 (0–37.50)	0.74**	–
SPAMDASH	0.97	19	25.00 (12.50–48.44)	25.00 (18.75–75.00)	0.92**	–

*Key*: Spearman’s correlations; ** *p* < 0.001

#### Test-retest reliability

Data for those participants reporting they were “the same” at Test 2 as at Test 1 were analysed. For all four DASH measures, correlations between Test 1 and Test 2 scores were strong (r_s_ = 0.74–0.95). For the DASH and QuickDASH, ICC(2,1) were excellent (see Table 5). As there are no Rasch transformation tables available for the WORK-and SPAMDASH, ICC(2,1) could not be calculated. For individual items in the DASH and QuickDASH, reliability was moderate (*n* = 9) or good (*n* = 21); for the WORKDASH moderate (*n* = 3) and good (*n* = 1); and SPAMDASH for all four items were good. (See Additional file 1: Table S2).

#### Sensitivity to change

Using Rasch transformed scores, for the DASH, SEM = 1.78 and MDC_95_ = 4.94; and Quick DASH SEM = 1.65 and MDC_95_ = 4.57. As there are no Rasch transformation tables available for the WORK-AN|D SPAMDASH, SEM and MDC_95_ could not be calculated.

#### Missing data

All 30 DASH items were answered by 226/340 (67%). One item was unanswered by 76 participants (23%); two by 20 (7%); three items by 4 (1%); and five items by 4 (1%). Three participants (1%) returned the DASH uncompleted. Scores could not be generated because of missing data for the following: DASH, 11 participants (3%); QuickDASH, 3 participants (< 1%); WORKDASH, 4 participants (2%); and SPAMDASH, two participants (3%). There were no significant differences in the characteristics, disease activity, symptom, function or quality of life scores of those for whom any DASH scores could be completed or not. However, those participants with missing data were more likely to be older (65.27 (SD 10.49) years vs 60.28 (SD 12.50) years, *t* = 3.66; *p* < 0.001); and to be single, divorced/separated or widowed/widowered (chi-square 9.25; df = 3; *p* = 0.03). Items unanswered by more than 5% of participants were: sexual activities (*n* = 56 (16%)); and recreational activities requiring little effort (*n* = 18 (5%)). Those not answering the sexual activities item were significantly: older (67.25 (SD10.25) years vs 60.91 (SD 12.15) years; *t* = 3.65; p < 0.001); and more likely to be living alone (chi-square 15.65, df = 1; p < 0.001) than those who did answer. This therefore reflected which participants were most likely to have missing data, as sexual activities was the commonest unanswered question.

#### Floor and ceiling effects

There were no floor or ceiling effects for the DASH (2% scored 0; 0.3% scored 100) or the QuickDASH (5.6% scored 0, 0% scored 100). However, for the WORK- and SPAM-DASH there were floor effects: 21 and 17.5% respectively. There were no ceiling effects for the WORKDASH (2%) but there were for the SPAMDASH (15.8%).

## Discussion

Linguistically validated British English versions of the DASH and QuickDASH are now available for use in the UK. These British-English translations demonstrated good psychometric properties in a sample of people with RA and can be used in both clinical practice and research.

We ensured linguistic and cross-cultural validity of the DASH by using the IWH DASH translation process, while gaining the developers’ approval throughout. During cognitive debriefing, some participants were unsure if “ability to do the following activities…” referred to ability with or without aids and adaptations, as ability can differ when using these. Clarifying this, to ensure respondents answer in the same way, could be beneficial. However, the 50 language versions currently available do not specify this, so these changes were not made.

In terms of content validity, the DASH scales address some of the Body Functions and over half of the Activities and Participation items in the Brief ICF Core Set for RA and those not covered by the DASH are mostly those not relevant to the arm, shoulder and hand. Some core issues are potentially relevant and not reflected in the DASH. These include: body image (1801), as many people can be disturbed by their hand appearance in RA [52]; muscle endurance (b740) and maintaining a body position (d415), as DASH ICF linking did not specifically identify prolonged and/or static actions [27]; and using communication devices and techniques (d360), as the use of smart/mobile phones and computers/tablets is now ubiquitous, compared to when the DASH was developed in 1995. However, participants did not raise such issues in the cognitive debriefing interviews suggesting the DASH adequately reflects their main problems. As device use is a common source of upper limb pain in those with high-frequency use, it may be time to update the DASH and include this as a new item, thus reflecting modern-day life. Potentially, it could replace an existing item which is now less common, e.g. change a lightbulb overhead, as the advent of LED bulbs means this activity is now less frequently performed.

Concurrent validity of the DASH scales was strong for the DASH, QuickDASH and WORKDASH and moderate to strong for the SPAMDASH, which may have been affected by the small sample size. Psychometric testing in RA has been conducted in three other language versions of the DASH in RA (Swedish, Turkish and Dutch) [6, 53, 54]. Results of the test-retest reliability indicate the DASH and Quick DASH can be used for both group and individual measurement in RA. Additionally, sensitivity to change (MDC_95_) indicated DASH and QuickDASH changes of about 5 (on a 0–100 scale) are similar to those reported by Kennedy et al. [11]. However, the MDC_95_ for the WORK- and SPAM-DASH could not be calculated as we do not have Rasch transformation tables available for these two modules. Rasch analysis also identified that the DASH and QuickDASH can be considered unidimensional and thus summed or standardised scores can be used [Prodinger B, Hammond A, Tennant A, Prior Y, Tyson S. Deconstructing the Disabilities of the Arm, Shoulder and Hand (DASH) and QuickDASH in Rheumatoid Arthritis, submitted]. A strength of this study is that we had a large sample of people with RA recruited from a wide variety of rheumatology out-patient clinics, meaning the results are representative for people with RA.

The limitations of this study are that we only tested the DASH and QuickDASH in people with RA. Further testing is recommended in other upper limb conditions to investigate psychometric properties. Responsiveness (i.e. longitudinal validity) still needs to be tested and minimal clinically important differences (MCID) also need to be established. Construct validity of the WORKDASH and SPAMDASH using Rasch analysis is also warranted.

## Conclusions

Overall, psychometric testing of the British English versions of the DASH, QuickDASH, WORKDASH and SPAMDASH demonstrated good validity and reliability in a British English speaking sample of people with RA in the UK. These four British English DASH scales meet most of the recommendations of the Consensus-based Standards for the selection of health Measurement Instruments (COSMIN) checklist [22, 55]. Accordingly, the British English DASH, QuickDASH, WORK-and SPAMDASH can be used in clinical practice and research in the UK and are available from the Institute of Work and Health DASH website [56, 57].

## Additional files

Additional file 1: **Table S1.** Linking between Brief ICF Core Set for Rheumatoid Arthritis (RA) and the DASH. **Table S2.** Test retest reliability for the DASH (*n* = 170), WORKDASH (*n* = 53) and SPAMDASH (*n* = 19) items (linear weighted kappas). (DOCX 41 kb)

## Abbreviations

DASH: Disabilities of the Arm, Shoulder and Hand Questionnaire
HAQ: Health Assessment Questionnaire
ICC: Intra-class correlation coefficient
ICF: International Classification of Functioning, Disability and Health
MAPHAND: Measure of activity performance of the hand questionnaire
MDC: Minimum detectable change
NRS: Numeric rating scale
PROM: Patient reported outcome measure
RA: Rheumatoid arthritis
RAQOL: Rheumatoid arthritis quality of life scale
SF36v2: Medical outcomes survey 36 item short form health survey version 2
SPAMDASH: Sport and music module of the disabilities of the arm, shoulder and hand questionnaire
WORKDASH: Work module of the disabilities of the arm, shoulder and hand questionnaire
