# Supplementary material for: Linguistic validation, validity and reliability of the British English versions of the Disabilities of the Arm, Shoulder and Hand (DASH) questionnaire and QuickDASH in people with rheumatoid arthritis
Source: BMC Musculoskelet Disord. 2018 Apr 16;19:118. doi: 10.1186/s12891-018-2032-8 (PMC5902839; doi:10.1186/s12891-018-2032-8)
Supplement: Supplementary file 1 — Table S1. Linking between Brief ICF Core Set for Rheumatoid Arthritis (RA) and the DASH. Table S2. Test retest reliability for the DASH (n = 170), WORKDASH (n = 53) and SPAMDASH (n = 19) items (linear weighted kappas). (DOCX 41 kb) [file 12891_2018_2032_MOESM1_ESM.docx]

**Table S1: Linking between Brief ICF Core Set for Rheumatoid Arthritis (RA) and the DASH.**

| Brief Core Set for RA ICF Components, Codes, Categories and Titles: | DASH Item (and ICF sub-codes if applicable) |
| --- | --- |
| 1 BODY FUNCTIONS: |  |
| b130 Energy and drive functions |  |
| b134 Sleep functions | 29. During the past week, how much difficulty have you had in sleeping because of the pain in your arm, shoulder or hand |
| b152 Emotional functions |  |
| b180 Experience of self and time functions |  |
| b1801 Body image |  |
| b2800 Generalized pain |  |
| b2801 Pain in body part |  |
| b28010 Pain in head and neck |  |
| b28013 Pain in back |  |
| b28014 Pain in upper limb | 24 Arm, shoulder or hand pain  25 Arm, shoulder or hand pain when performing specific activities |
| b28015 Pain in lower limb |  |
| b28016 Pain in joints | 24 Arm, shoulder or hand pain  25 Arm, shoulder or hand pain when performing specific activities. |
| b430 Haematological system functions |  |
| b510 Ingestion functions |  |
| b640 Sexual functions |  |
| b7102 Mobility of joints generalized | 19 Recreational activities in which you move your arm freely (e.g. playing Frisbee, badminton etc) |
| b715 Stability of joint functions |  |
| b740 Muscle endurance functions |  |
| b770 Gait pattern functions |  |
| b7800 Sensation of muscle stiffness | 28. Stiffness in your arm, shoulder or hand. |
| 2 BODY STRUCTURES: |  |
| s73001 Elbow joint |  |
| s73011 Wrist joint |  |
| s7302 Structure of hand |  |
| s73021 Joints of hand and fingers |  |
| s73022 Muscles of hand |  |
| s75001 Hip joint |  |
| s75011 Knee joint |  |
| s7502 Structure of ankle and foot |  |
| s760 Structure of trunk |  |
| s7600 Structure of vertebral column |  |
| s76000 Cervical vertebral column |  |
| s770 Additional musculoskeletal structures related to movement |  |
| s810 Structure of areas of skin |  |
| 3 ACTIVITIES AND PARTICIPATION: |  |
| d170 Writing | 2 Write |
| d360 Using communication devices and techniques |  |
| d415 Maintaining a body position |  |
| d430 Lifting and carrying objects | 10 Carry a shopping bag or briefcase (4301 Carrying in the hands)  11 Carry a heavy object (over 10lbs/5 kgs). (d4301) |
| d449 Carrying, moving and handling objects, other specified and unspecified | 1 Open a tight or new jar (d4453 turn or twist hands or arms)  3 Turn a key (d4453)  5 Push open a heavy door (d4451 Pushing)  6 Place an object on a shelf above your head (d4452 Reaching)  12 Change a light bulb overhead (d4453)  17 Recreational activities which require little effort (e.g. card playing), knitting etc.) (d4400 picking up; d4401 grasping; d4402 manipulating; d4403 releasing)  18 Recreational activities in which you take some force or impact through your arm, shoulder or hand (e.g. golf, hammering, tennis etc.) (d4454 Throwing) |
| d455 Moving around |  |
| d460 Moving around in different locations |  |
| d465 Moving around using equipment |  |
| d470 Using transportation | 20 Manage transport needs (getting from one place to another) |
| d475 Driving | 20 Manage transport needs (getting from one place to another) (d4751 driving motorized vehicles) |
| d510 Washing oneself | 14 Wash your back (d5100 Washing body parts) |
| d520 Caring for body parts | 13 Wash or blow dry your hair (d5202 Caring for hair) |
| d530 Toileting |  |
| d540 Dressing | 15 Put on a jumper (d5400 Putting on clothes) |
| d550 Eating | 16 Use a knife to cut food |
| d560 Drinking |  |
| d570 Looking after one`s health |  |
| d620 Acquisition of goods and services |  |
| d630 Preparing meals | 4 Prepare a meal |
| d640 Doing housework | 7 Do heavy household jobs (e.g. wash windows, clean floors) (d6402 Clean living area)  9 Make a bed |
| d660 Assisting others |  |
| d760 Family relationships | 22 During the past week, to what extent has your arm, shoulder or hand problem interfered with your normal social activities with family (d760), friends, neighbours or groups? |
| d770 Intimate relationships | 21 Sexual activities (d7702 sexual relationships) |
| d859 Work and employment, other specified and unspecified | 23 During the past week, were you limited in your work or other regular daily activities as a result of your arm, shoulder or hand problem? |
| d910 Community life |  |
| d920 Recreation and leisure | 17 Recreational activities which require little effort (e.g. card playing, knitting etc.) (d920: cards (d9200 Play); knitting (d9203 Crafts).  18 Recreational activities in which you take some force or impact through your arm, shoulder or hand (e.g. golf, hammering, tennis etc) (d9201 Sports; d9204 Hobbies)  19 Recreational activities in which you move your arm freely (e.g. playing Frisbee, badminton etc) (d9201 Sports) |
| 4. ENVIRONMENTAL FACTORS: |  |
| e115 Products and technology for personal use in daily living |  |
| e310 Immediate family |  |
| e355 Health Professionals |  |
| e570 Social security services, systems and policies |  |
| e580 Health services, systems and policies |  |
| DASH items not included in the Brief ICF Core Set for RA: | |
| ICF Code, Category and Title: | **DASH item** |
| b265 Touch function | 26 Tingling (pins and needles) in your arm, shoulder or hand. |
| b730 Muscle power functions* | 27 Weakness in your arm, shoulder or hand. |
| d650 Caring for household objects | 8 Garden or outdoor property work (d6505 Taking care of plants, indoors and outdoors) |
| d750 Informal social relationships | 22 During the past week, to what extent has your arm, shoulder or hand problem interfered with your normal social activities with family, friends (d7500 informal relationships with friends), neighbours (d7501 informal relationships with neighbours), or groups (d7504 informal relationships with peers)? |
| personal factor (pf) | 30 I feel less capable, less confident or less useful because of my arm, shoulder or hand problem. |

Key: * item is included in Comprehensive ICF Core Set for RA.

Table S2: Test retest reliability for the DASH (n=170), WORKDASH (n=53) and SPAMDASH (n=19) items (linear weighted kappas).

| **DASH1: Open jar**   \| Weighted Kappa \| 0.652 \| \| --- \| --- \| \| Standard error \| 0.0307 \| \| 95% CI \| 0.592 to 0.712 \|   **DASH2: Write**   \| Weighted Kappa \| 0.717 \| \| --- \| --- \| \| Standard error \| 0.0326 \| \| 95% CI \| 0.654 to 0.781 \|   **DASH3: Turn a key**   \| Weighted Kappa \| 0.679 \| \| --- \| --- \| \| Standard error \| 0.0320 \| \| 95% CI \| 0.616 to 0.742 \|   **DASH4: Prepare meal**   \| Weighted Kappa \| 0.732 \| \| --- \| --- \| \| Standard error \| 0.0310 \| \| 95% CI \| 0.671 to 0.793 \|   **DASH5: Open heavy door**   \| Weighted Kappa \| 0.682 \| \| --- \| --- \| \| Standard error \| 0.0295 \| \| 95% CI \| 0.624 to 0.74 \|   **DASH16: Use knife to cut food**   \| Weighted Kappa \| 0.609 \| \| --- \| --- \| \| Standard error \| 0.0324 \| \| 95% CI \| 0.546 to 0.673 \|   **DASH17: Recreational activities – little effort**   \| Weighted Kappa \| 0.579 \| \| --- \| --- \| \| Standard error \| 0.0376 \| \| 95% CI \| 0.505 to 0.653 \|   **DASH18: Recreational activities – force or impact**   \| Weighted Kappa \| 0.693 \| \| --- \| --- \| \| Standard error \| 0.0275 \| \| 95% CI \| 0.639 to 0.747 \|   **DASH19: Recreational activities move arm freely**   \| Weighted Kappa \| 0.641 \| \| --- \| --- \| \| Standard error \| 0.0322 \| \| 95% CI \| 0.578 to 0.704 \|   **DASH20: Manage transport needs**   \| Weighted Kappa \| 0.663 \| \| --- \| --- \| \| Standard error \| 0.0334 \| \| 95% CI \| 0.597 to 0.728 \| | **DASH6: Place object on shelf**   \| Weighted Kappa \| 0.690 \| \| --- \| --- \| \| Standard error \| 0.0257 \| \| 95% CI \| 0.64 to 0.74 \|   **DASH7: Heavy household jobs**   \| Weighted Kappa \| 0.712 \| \| --- \| --- \| \| Standard error \| 0.0265 \| \| 95% CI \| 0.66 to 0.764 \|   **DASH8: Garden/ property work**   \| Weighted Kappa \| 0.722 \| \| --- \| --- \| \| Standard error \| 0.0261 \| \| 95% CI \| 0.671 to 0.773 \|   **DASH9: Make bed**   \| Weighted Kappa \| 0.648 \| \| --- \| --- \| \| Standard error \| 0.0307 \| \| 95% CI \| 0.588 to 0.708 \|   **DASH10: Carrying shopping bag**   \| Weighted Kappa \| 0.611 \| \| --- \| --- \| \| Standard error \| 0.0320 \| \| 95% CI \| 0.548 to 0.673 \|   **DASH21: Sexual activities**   \| Weighted Kappa \| 0.769 \| \| --- \| --- \| \| Standard error \| 0.0317 \| \| 95% CI \| 0.707 to 0.831 \|   **DASH22: Past week – norm social act**   \| Weighted Kappa \| 0.577 \| \| --- \| --- \| \| Standard error \| 0.0307 \| \| 95% CI \| 0.517 to 0.637 \|   **DASH23: Past week – limited in work/ daily act**   \| Weighted Kappa \| 0.656 \| \| --- \| --- \| \| Standard error \| 0.0299 \| \| 95% CI \| 0.598 to 0.715 \|   **DASH24: Arm, shoulder, hand pain**   \| Weighted Kappa \| 0.596 \| \| --- \| --- \| \| Standard error \| 0.0342 \| \| 95% CI \| 0.529 to 0.663 \|   **DASH25: Arm, shoulder, hand pain – specific activity**   \| Weighted Kappa \| 0.540 \| \| --- \| --- \| \| Standard error \| 0.0368 \| \| 95% CI \| 0.467 to 0.612 \| | **DASH11: Carry heavy object**   \| Weighted Kappa \| 0.699 \| \| --- \| --- \| \| Standard error \| 0.0288 \| \| 95% CI \| 0.642 to 0.755 \|   **DASH12: Change a light bulb**   \| Weighted Kappa \| 0.728 \| \| --- \| --- \| \| Standard error \| 0.0252 \| \| 95% CI \| 0.678 to 0.777 \|   **DASH13: Wash/ blow hair**   \| Weighted Kappa \| 0.723 \| \| --- \| --- \| \| Standard error \| 0.0285 \| \| 95% CI \| 0.667 to 0.779 \|   **DASH14: Wash back**   \| Weighted Kappa \| 0.693 \| \| --- \| --- \| \| Standard error \| 0.0283 \| \| 95% CI \| 0.637 to 0.748 \|   **DASH15: Put on jumper**   \| Weighted Kappa \| 0.653 \| \| --- \| --- \| \| Standard error \| 0.0321 \| \| 95% CI \| 0.59 to 0.716 \|   **DASH26: Tingling in arm, shoulder, hand**   \| Weighted Kappa \| 0.543 \| \| --- \| --- \| \| Standard error \| 0.0385 \| \| 95% CI \| 0.467 to 0.618 \|   **DASH27: Weakness in arm, shoulder, hand**   \| Weighted Kappa \| 0.565 \| \| --- \| --- \| \| Standard error \| 0.0351 \| \| 95% CI \| 0.496 to 0.634 \|   **DASH28: Stiffness in arm, shoulder, hand**   \| Weighted Kappa \| 0.495 \| \| --- \| --- \| \| Standard error \| 0.0362 \| \| 95% CI \| 0.424 to 0.566 \|   **DASH29: Sleeping difficulty due to arm, shoulder, hand pain**   \| Weighted Kappa \| 0.572 \| \| --- \| --- \| \| Standard error \| 0.0349 \| \| 95% CI \| 0.503 to 0.64 \|   **DASH30: Capability/ confidence due to arm, shoulder, hand**   \| Weighted Kappa \| 0.564 \| \| --- \| --- \| \| Standard error \| 0.0344 \| \| 95% CI \| 0.497 to 0.632 \| |
| --- | --- | --- | --- | --- | --- | --- | --- | --- | --- | --- | --- | --- | --- | --- | --- | --- | --- | --- | --- | --- | --- | --- | --- | --- | --- | --- | --- | --- | --- | --- | --- | --- | --- | --- | --- | --- | --- | --- | --- | --- | --- | --- | --- | --- | --- | --- | --- | --- | --- | --- | --- | --- | --- | --- | --- | --- | --- | --- | --- | --- | --- | --- | --- | --- | --- | --- | --- | --- | --- | --- | --- | --- | --- | --- | --- | --- | --- | --- | --- | --- | --- | --- | --- | --- | --- | --- | --- | --- | --- | --- | --- | --- | --- | --- | --- | --- | --- | --- | --- | --- | --- | --- | --- | --- | --- | --- | --- | --- | --- | --- | --- | --- | --- | --- | --- | --- | --- | --- | --- | --- | --- | --- | --- | --- | --- | --- | --- | --- | --- | --- | --- | --- | --- | --- | --- | --- | --- | --- | --- | --- | --- | --- | --- | --- | --- | --- | --- | --- | --- | --- | --- | --- | --- | --- | --- | --- | --- | --- | --- | --- | --- | --- | --- | --- | --- | --- | --- | --- | --- | --- | --- | --- | --- | --- | --- | --- | --- | --- | --- | --- | --- | --- |

| **WM1: Work usual way**   \| Weighted Kappa \| 0.618 \| \| --- \| --- \| \| Standard error \| 0.0627 \| \| 95% CI \| 0.495 to 0.74 \|   **WM2: Arm shoulder hand pain**   \| Weighted Kappa \| 0.579 \| \| --- \| --- \| \| Standard error \| 0.0607 \| \| 95% CI \| 0.46 to 0.698 \|   **WM3: Working as well as I would like**   \| Weighted Kappa \| 0.587 \| \| --- \| --- \| \| Standard error \| 0.0605 \| \| 95% CI \| 0.469 to 0.706 \|   **WM4: Working usual amount of time**   \| Weighted Kappa \| 0.490 \| \| --- \| --- \| \| Standard error \| 0.0705 \| \| 95% CI \| 0.352 to 0.629 \| | **SPAM1: Play usual way**   \| Weighted Kappa \| 0.694 \| \| --- \| --- \| \| Standard error \| 0.102 \| \| 95% CI \| 0.493 to 0.894 \|   **SPAM2: Arm shoulder hand pain**   \| Weighted Kappa \| 0.716 \| \| --- \| --- \| \| Standard error \| 0.0807 \| \| 95% CI \| 0.558 to 0.874 \|   **SPAM3: Play as well as I would like**   \| Weighted Kappa \| 0.770 \| \| --- \| --- \| \| Standard error \| 0.0669 \| \| 95% CI \| 0.639 to 0.901 \|   **SPAM4: Playing usual amount of time**   \| Weighted Kappa \| 0.773 \| \| --- \| --- \| \| Standard error \| 0.0600 \| \| 95% CI \| 0.655 to 0.89 \| |  |
| --- | --- | --- | --- | --- | --- | --- | --- | --- | --- | --- | --- | --- | --- | --- | --- | --- | --- | --- | --- | --- | --- | --- | --- | --- | --- | --- | --- | --- | --- | --- | --- | --- | --- | --- | --- | --- | --- | --- | --- | --- | --- | --- | --- | --- | --- | --- | --- | --- | --- | --- |
